# Enhanced Delta-Notch Lateral Inhibition Model Incorporating Intracellular Notch Heterogeneity and Tension-Dependent Rate of Delta-Notch Binding that Reproduces Sprouting Angiogenesis Patterns

**DOI:** 10.1038/s41598-018-27645-1

**Published:** 2018-06-22

**Authors:** Yen Ling Koon, Songjing Zhang, Muhammad Bakhait Rahmat, Cheng Gee Koh, Keng-Hwee Chiam

**Affiliations:** 1 Interdisciplinary Graduate School, Nanyang Technological University, Singapore, Singapore; 2 A*STAR Bioinformatics Institute, Singapore, Singapore; 3 Mechanobiology Institute, National University of Singapore, Singapore, Singapore; 4 School of Biological Sciences, Nanyang Technological University, Singapore, Singapore

## Abstract

Endothelial cells adopt unique cell fates during sprouting angiogenesis, differentiating into tip or stalk cells. The fate selection process is directed by Delta-Notch lateral inhibition pathway. Classical Delta-Notch models produce a spatial pattern of tip cells separated by a single stalk cell, or the salt-and-pepper pattern. However, classical models cannot explain alternative tip-stalk patterning, such as tip cells that are separated by two or more stalk cells. We show that lateral inhibition models involving only Delta and Notch proteins can also recapitulate experimental tip-stalk patterns by invoking two mechanisms, specifically, intracellular Notch heterogeneity and tension-dependent rate of Delta-Notch binding. We introduce our computational model and analysis where we establish that our enhanced Delta-Notch lateral inhibition model can recapitulate a greater variety of tip-stalk patterning which is previously not possible using classical lateral inhibition models. In our enhanced Delta-Notch lateral inhibition model, we observe the existence of a hybrid cell type displaying intermediate tip and stalk cells’ characteristics. We validate the existence of such hybrid cells by immuno-staining of endothelial cells with tip cell markers, Delta and CD34, which substantiates our enhanced model.

## Introduction

During sprouting angiogenesis, endothelial cells form sprouts that grow towards an angiogenic stimulus. Two distinct phenotypes are undertaken by the endothelial cells in the nascent blood vessel sprout, namely the tip cell phenotype and the stalk cell phenotype^1,2^. Tip cells are defined by their long fingerlike protrusions called filopodia which bring about motile behaviour. These cells migrate towards the angiogenic source upon stimulation by chemotactic factors^3^. The second type of cells known as stalk cells trail behind the tip cells in the growing sprout. Stalk cells support the growth of the vessel by their proliferative capacity. In addition, stalk cells ensure stability and integrity of the young sprout by forming adherent and tight junctions^1^.

**Figure 1:**
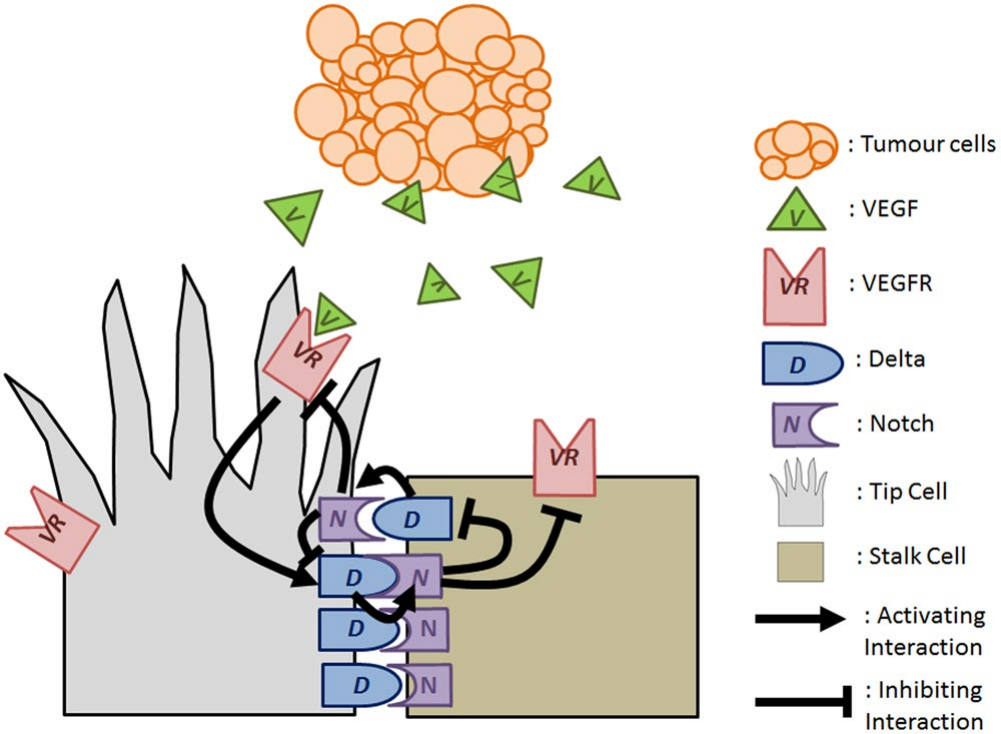
Schematic of Delta-Notch Lateral Inhibition. Tumour cells secrete angiogenic factors such as vascular endothelial growth factor (VEGF). VEGF binds to VEGF-receptor (VEGFR) on the surfaces of endothelial cells leading to the activation of VEGFR. Activated VEGFR causes upregulation of transmembrane ligand, Delta. Delta ligand binds to the transmembrane receptor, Notch of its neighbouring cell. Upon Delta ligand binding, Notch of the neighbouring cell becomes activated and inhibits VEGFR and Delta expression.

How an endothelial cell becomes tip cell or stalk cell is through the Delta-Notch lateral inhibition process^2,4^. In essence, lateral inhibition prevents the neighbours of a tip cell from taking on the same fate as itself. One of the more commonly known angiogenic factors is the vascular endothelial growth factor, VEGF^5^. VEGF binds to VEGF-receptor (VEGFR) on the surfaces of endothelial cells thereby activating VEGFR. Activated VEGFR goes on to increase expression of Delta-like ligand 4, here and so forth termed as Delta. Delta is a transmembrane ligand which binds to the transmembrane receptor, Notch of its neighbouring cell. Upon ligand binding, Notch becomes activated and undergoes proteolytic cleavage. The cleaved intracellular domain of Notch (NICD) can translocate to the nucleus to modulate gene expression. The cascade of signaling events ultimately culminates in down regulation of VEGFR and Delta^6–8^. The aforementioned signalling activities are depicted in Fig. 1. As a result, a high Delta cell which has low Notch acitivity will have a low Delta, high Notch cell as its neighbour. Tip cells are characterized by a high Delta, low Notch expression while stalk cells are defined by a low Delta, high Notch expression. Lateral inhibition thus prevents the neighbours of a tip cell from attaining the same tip cell fate. Such regulation is of marked importance. If all cells become tip cells, the blood vessel will fall apart. On the other hand, if all cells become stalk cells, the blood vessel can only grow in diameter and not in length^9^. Lateral inhibition thus tunes the proportion of tip and stalk cells for optimal growth and cohesion of the blood vessel.

**Figure 2:**
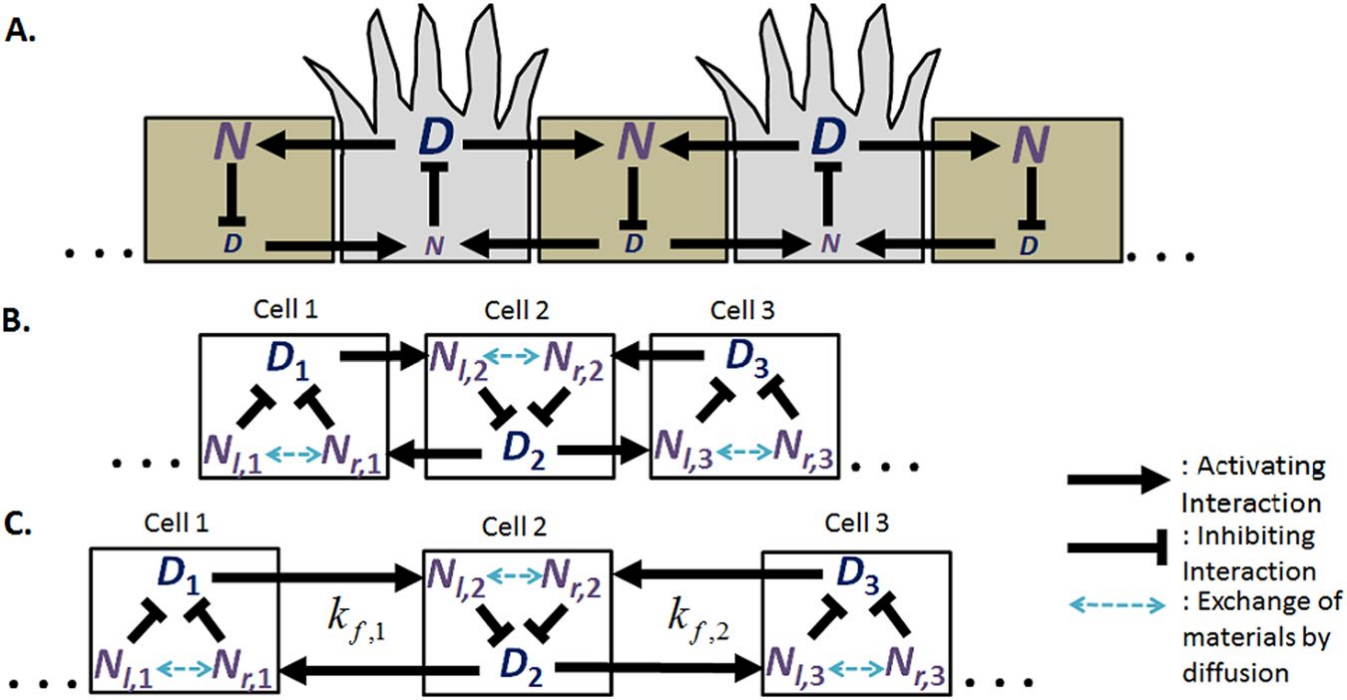
Lateral Inhibition Models. (**A**) Classical Delta-Notch lateral inhibition models describe the following reactions: Delta of one cell binds to Notch of the neighbouring cell, and Notch inhibits Delta expression within the same cell. Computational models of classical Delta-Notch lateral inhibition yield the salt-and-pepper pattern where tip cells are separated exactly by one stalk cell. (**B**) Schematic of lateral inhibition model with intracellular Notch heterogeneity. (**C**) Schematic of lateral inhibition model with intracellular Notch heterogeneity and tension-dependent rate of Delta-Notch binding. In lateral inhibition model with intracellular Notch heterogeneity and tension-dependent rate of Delta-Notch binding, rate constants are a function of the adherent Delta-Notch pairs.

Classical lateral inhibition models predict a salt-and-pepper pattern in which tip cells are separated by one stalk cell as illustrated in Fig. 2A^10,11^. However, other angiogenic patterns where tip cells are separated by more than one stalk cell have been observed both *in vitro* and *in vivo*. The existence of two and three stalk cells spaced between tip cells can be seen from whole mounts of mouse retinas^12,13^. Several lateral inhibition models involving only Delta and Notch proteins have been proposed to explain such patterns. Most of these models seek to increase the number of cellular states possible such that cells are no longer limited to a high Delta-low Notch state or a low Delta-high Notch state. To accomplish this, Collier *et al*. proposed a two-dimensional grid system where the increased number of cell-cell contacts allow for creation of cells with moderate Delta and Notch levels^11^. These moderate cells can accommodate a larger number of stalk cells spaced between tip cells^11^. Similarly, Cohen *et al*. proposed a modified lateral inhibition model where each cell contacts both its direct neighbours as well as their neighbours’ neighbours to account for bristle spacing observed within the *Drosophila* dorsal thorax^14^. In the latter model, the increase in cell contacts are brought about by the presence of dynamic filopodia^14^. Lastly, Chen *et al*. included a mechanism involving a nearest-neighbour Notch gradient to reproduce patterning of epidermal sensory neurons within *Ciona intestinalis*^15^. Even though these models can resolve the patterns observed in their respective cell types, they have their shortcomings in explaining tip-stalk patterns observed in sprouting angiogenesis. Angiogenic sprouts are typically one-dimensional in nature which defies the two-dimensional system of Collier’s^11^. Cohen’s model^14^ requires interaction between the filpodia of the tip cell and the stalk cell. This is contrasted during angiogenesis where the filopodia of tip cells guides the angiogenic sprout towards the direction of migration^3^ and are typically not observed to interact with the lagging stalk cells. Lastly, the Notch gradient term in Chen’s^15^ has so far not being observed experimentally.

Most models that simulate the Delta-Notch pathway makes the inherent assumption that Delta and Notch levels are homogeneous within the cell. Hence, we wonder if inclusion of differential localization of Notch into the classical lateral inhibition model will reproduce the various angiogenic patterns observed. We discover that by considering intracellular Notch heterogeneity, more cellular states can be attained. With these additional states, a limited set of angiogenic patterns can be created where tip cells are separated by more than one stalk cell. Certain types of patterning are however never observed such as the case where three stalk cells are found between a pair of tip cells. This is due to inherent symmetry within the system of cells that invariably reduces a three-stalk cell spacing pattern to a one-stalk cell spacing pattern (will be discussed in detail in Results).

Mechanical forces has been shown to modulate responses between ligand-receptor binding^16,17^. Such force modulation has also been observed in Delta-Notch signalling where the rate of ligand-receptor binding is dependent on the adhesive strength between cells^18^. Intercellular adhesion or tension impacts signalling by modifying the binding reaction between ligand and receptor. It is currently unknown how adhesion or tension between cells regulate Delta-Notch signalling and therefore tip-stalk pattering. We seek to examine if tension-dependent rate of Delta-Notch binding can be the symmetry-breaking mechanism that is necessary on top of intracellular Notch heterogeneity to recreate tip-stalk patterns seen during angiogenesis.

In this paper, we aim to elucidate the role of intracellular Notch heterogeneity and tension-dependent rate of Delta-Notch binding, two commonly overlooked mechanisms, on Delta-Notch signalling. These mechanisms would be pervasive in all cells expressing Delta and Notch. Unfortunately, it is currently unknown how these mechanisms affect Delta and Notch’s regulation. We investigate if addition of intracellular Notch heterogeneity and tension-dependent rate of Delta-Notch binding into the classical lateral inhibition model will explain and recapitulate the various forms of tip-stalk patterning observed during sprouting angiogenesis.

## Results

### Tip-Stalk Patterns with More Than One Stalk Cell in between Tip Cells Recovered in Lateral Inhibition Model with Intracellular Notch Heterogeneity

In ths section, we first present the results where we consider intracellular Notch heterogeneity in the lateral inhibition model without tension-dependent rate of Delta-Notch binding, i.e. *h* = 0 in Eq. (20). (Since *h* = 0, *k*_*f*,*j*_ = *k*_*f*0_ for all cells). In most classical lateral inhibition models, Notch heterogeneity is often not studied^11,19,20^. It is currently unknown how a heterogeneous intracellular concentration of Notch will affect tip-stalk patterning.

Without Notch heterogeneity, the classical lateral inhibition model yields two forms of patterning. The first pattern is made up of a uniform array of cells each consisting of identical Delta and Notch levels. In the first pattern, no clear tip or stalk cell is present since all cells are identical. This is contrasted with the second form of patterning which produces the salt-and-pepper configuration, where the cells adopt an alternating arrangement of tip and stalk cells. Tip cells are defined by high Delta expression while stalk cells of high Notch. The first pattern where a single cellular phenotype is observed can be attributed to the existence of a lone stable steady state which becomes unstable at increasing nonlinearity leading to its eventual unstability. On the other hand, two stable states emerge in this nonlinear regime. These two stable states correspond to the high Delta-low Notch state and the low Delta-high Notch state responsible for the salt-and-pepper configuration.

**Figure 3:**
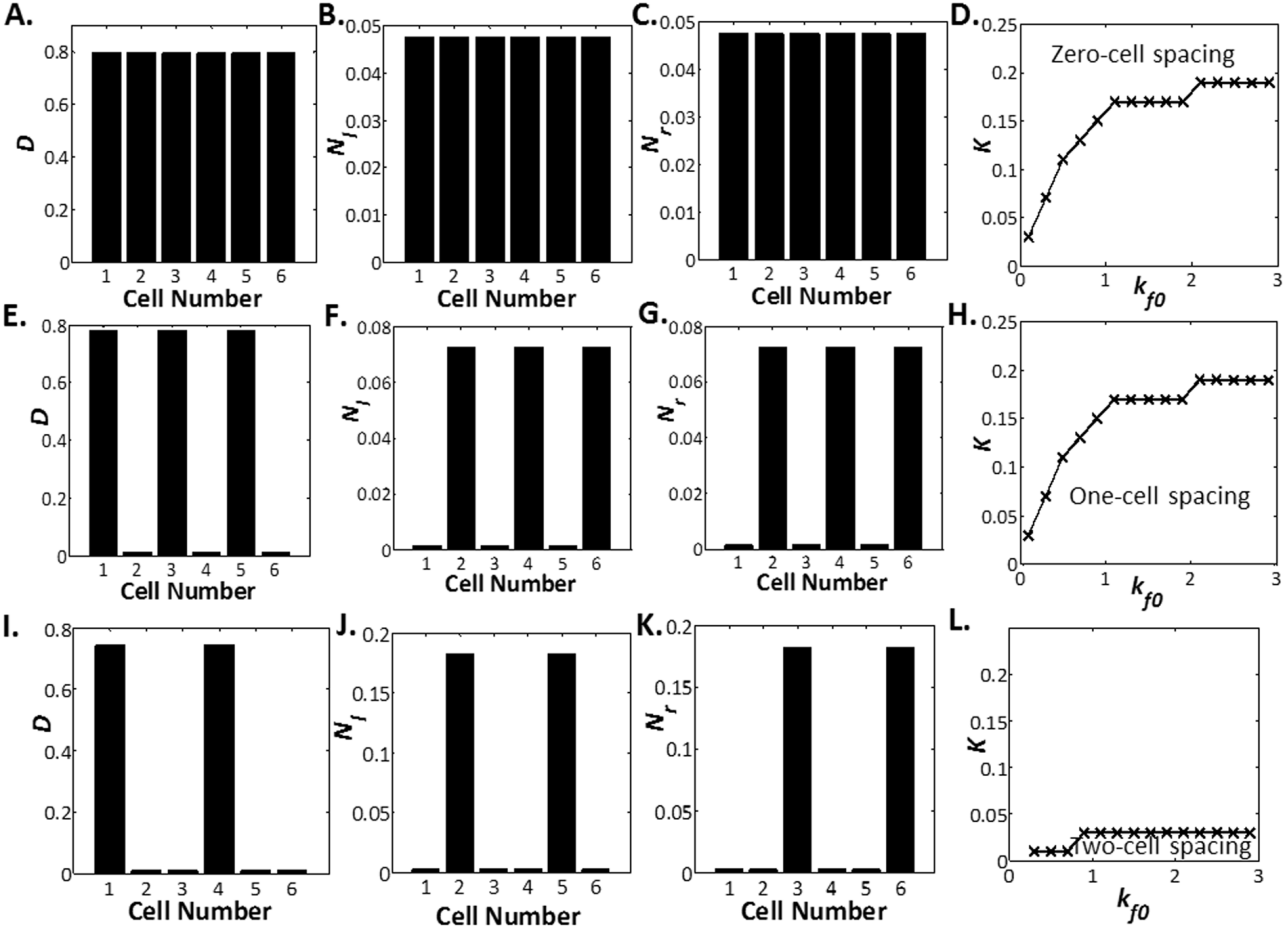
Delta and Notch Levels in Lateral Inhibition with Intracellular Notch Heterogeneity. Delta levels (**A**), Notch-left levels (**B**) and Notch-right levels (**C**) plotted against cell number for zero-cell spacing at *h* = 0, *W* = 0, *b*_0_ = 0.5, *K* = 1, *k*_*f*0_ = 0.1 and *k*_*d*_ = 1. (**D**) Parameter space of *K* vs *k*_*f*0_ where zero-cell spacing is observed at *h* = 0, *W* = 0, *b*_0_ = 0.8 and *k*_*d*_ = 0.1. Delta levels (**E**), Notch-left levels (**F**) and Notch-right levels (**G**) plotted against cell number for one-cell spacing at *h* = 0, *W* = 0, *b*_0_ = 0.8, *K* = 0.01, *k*_*f*0_ = 0.1 and *k*_*d*_ = 1. (**H**) Parameter space of *K* vs *k*_*f*0_ where one-cell spacing is observed at *h* = 0, *W* = 0, *b*_0_ = 0.8 and *k*_*d*_ = 0.1. Delta levels (**I**), Notch-left levels (**J**) and Notch-right levels (**K**) plotted against cell number for two-cell spacing at *h* = 0, *W* = 0, *b*_0_ = 0.8, *K* = 0.01, *k*_*f*0_ = 0.3 and *k*_*d*_ = 1. (**L**) Parameter space of *K* vs *k*_*f*0_ where two-cell spacing is observed at *h* = 0, *W* = 0, *b*_0_ = 0.8 and *k*_*d*_ = 0.1. For cell 2, 3, 5 and 6, the Notch-left levels and Notch-right levels are different.

When we consider intracellular Notch heterogeneity with lateral inhibition, we similarly recover the cellular states responsible for the salt-and-pepper configuration, namely, the high Delta-low Notch state and the low Delta-high Notch state. Correspondingly, like the classical lateral inhibition case, the salt-and-pepper patterning or the one-cell spacing pattern is observed at high nonlinearity characterized by high *k*_*f*0_ and low *K*, as depicted in Fig. 3H. A high *k*_*f*0_ in Eqs (15) and (16) indicates a lower level of Delta necessary for activation of the neighbouring Notch receptor. On the other hand, a low *K* in Eq. (14) signifies a lower concentration of activated Notch necessary for maximal inhibition of Delta.

We also uncover other forms of patterning previously unseen in classical lateral inhibition models such as the two-cell spacing. Parameter space for two-cell spacing is demarcated in Fig. 3L. These forms of patterning exist due to the creation of additional cellular states when Notch is allowed to exhibit intracellular heterogeneity. For example, a stable cellular state with moderate Delta levels, high Notch on one side and low Notch on the other can exist when we consider Notch heterogeneity in lateral inhibition. Examination of the two-cell spacing case in Fig. 3I–K reveals that the two stalk cells spaced between the tip cells exhibit the aforementioned characteristics: high Notch on one side and low Notch on the other.

**Figure 4:**
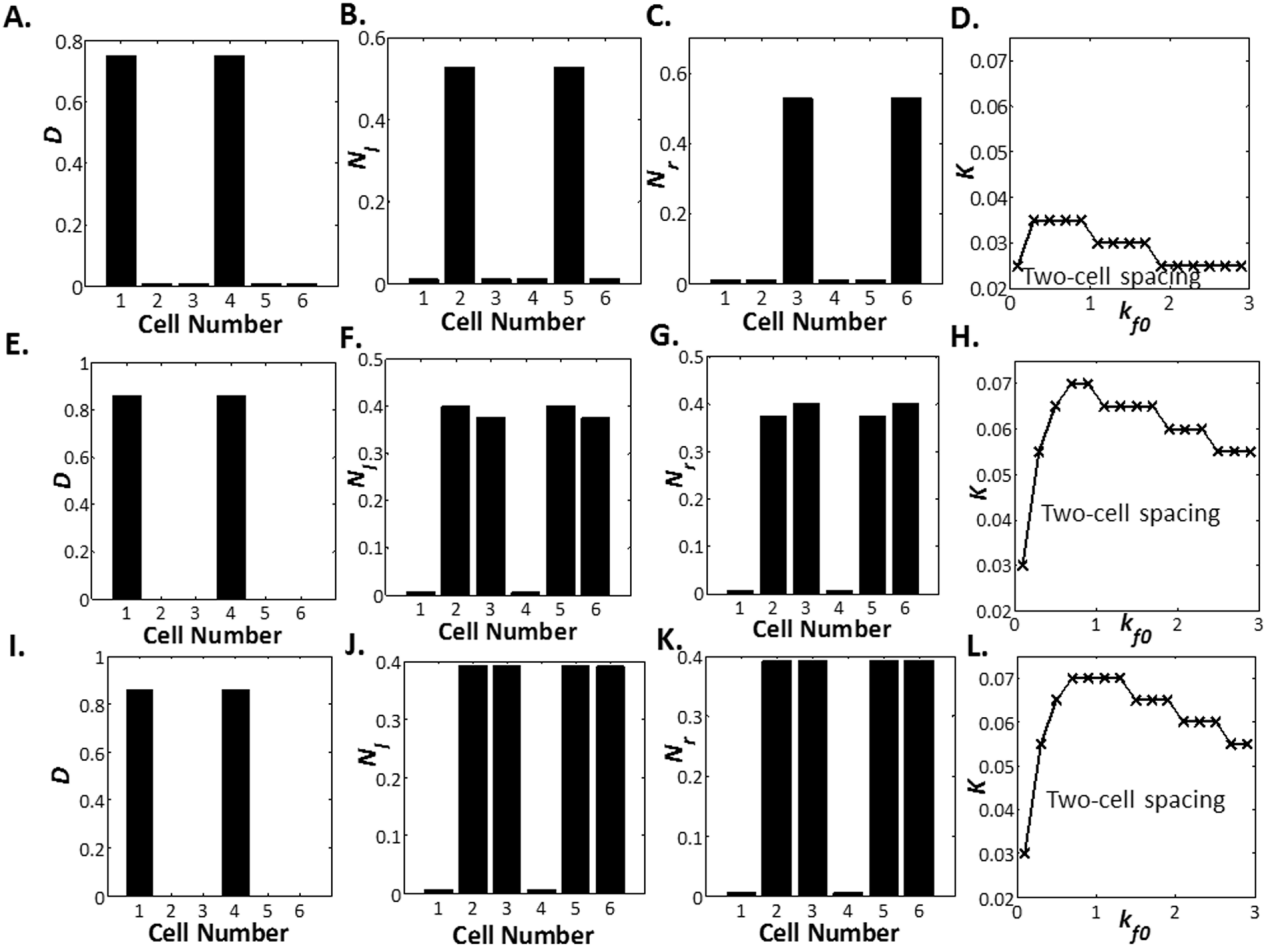
Effect of Diffusion on Three-cell Spacing for Lateral Inhibition with Intracellular Notch Heterogeneity. Delta levels (**A**), Notch-left levels (**B**) and Notch-right levels (**C**) plotted against cell number for two-cell spacing at *h* = 0, *W* = 0, *b*_0_ = 0.9, *K* = 0.025, *k*_*f*0_ = 0.3 and *k*_*d*_ = 0.2. (**D**) Parameter space of *K* vs *k*_*f*0_ where two-cell spacing is observed at *h* = 0, *W* = 0, *b*_0_ = 0.9 and *k*_*d*_ = 0.2. Delta levels (**E**), Notch-left levels (**F**) and Notch-right levels (**G**) plotted against cell number for two-cell spacing at *h* = 0, *W* = 3, *b*_0_ = 0.9, *K* = 0.025, *k*_*f*0_ = 0.3 and *k*_*d*_ = 0.2. (**H**) Parameter space of *K* vs *k*_*f*0_ where two-cell spacing is observed at *h* = 0, *W* = 3, *b*_0_ = 0.9 and *k*_*d*_ = 0.2. Delta levels (**I**), Notch-left levels (**J**) and Notch-right levels (**K**) plotted against cell number for two-cell spacing at *h* = 0, *W* = 50, *b*_0_ = 0.9, *K* = 0.025, *k*_*f*0_ = 0.3 and *k*_*d*_ = 0.2. (**L**) Parameter space of *K* vs *k*_*f*0_ where two-cell spacing is observed at *h* = 0, *W* = 50, *b*_0_ = 0.9 and *k*_*d*_ = 0.2.

Next, we examine how patterning is affected by intracellular diffusion by varying the value of *W*. Since the Notch-left levels and the Notch-right levels are identical in the zero-cell spacing and the one-cell spacing, parameter space for zero-cell spacing and one-cell spacing is independent of diffusion. The same is not true for the two-cell spacing pattern. We plot the parameter regimes where the two-cell spacing can be identified under a range of *W* in Fig. 4. Intriguingly, we observe that as long as diffusion remains finite, it is always possible to have a stable steady state solution for two-cell spacing. More details can be found in the Supplementary Information.

Even though we observe more tip-stalk patterns after we consider intracellular Notch heterogeneity, some cell spacings are never observed such as the three-cell spacing. We illustrate why below.

The equations for *N*_1_ and *N*_3_ in the three-cell spacing case at steady state is as follows:

1 $$0=-\,{k}_{d}{N}_{1}+{k}_{f}({D}_{2}\mathrm{)(1}-{N}_{1})$$

2 $$0=-\,{k}_{d}{N}_{3}+{k}_{f}({D}_{2}\mathrm{)(1}-{N}_{3})$$

Rearranging:

3 $${N}_{1}=\frac{{k}_{f}{D}_{2}}{{k}_{d}+{k}_{f}{D}_{2}}$$

4 $${N}_{3}=\frac{{k}_{f}{D}_{2}}{{k}_{d}+{k}_{f}{D}_{2}}$$

Thus, *N*_1_ is equal to *N*_3_, and consequently, *D*_1_ and *D*_3_ are identical to each other. As such, the three-cell spacing will never be observed in lateral inhibition if we only consider intracellular Notch heterogeneity. This is because, the three-cell spacing case will be reduced to the one-cell spacing pattern after symmetry considerations.

All in all, by considering intracellular Notch heterogeneity, we are able to reproduce more forms of tip-stalk patterning than that observed from classical lateral inhibition models. Nonetheless, some patterns such as the three-cell spacing cannot exist due to implicit symmetry constraints.

### Tip-Stalk Patterns with More Than One Stalk Cell in between Tip Cells Recovered in Lateral Inhibition Model with Tension-Dependent Rate of Delta-Notch Binding

Delta and Notch being a transmembrane receptor-ligand pair, has been implicated in cell-cell adhesion. When Notch or Delta function is reduced, cell adhesion is observed to decrease^21–24^. Morever, when cells exclusively expressing Notch are mixed with cells solely expressing Delta, large cell aggregates are observed^25^. This implies that Notch and Delta proteins besides playing a role in signalling, may also perform adhesive functions. At the same time, atomic force microscopy experiments have shown that Delta pulling accelerates Notch signalling^18^. This together with analysis of the Notch receptor structure have established a model for processing of Notch. In this model, pulling of the ligand-receptor pair triggers the receptor to unfold thereby unmasking an ADAM (a disintegrin and metalloprotease) cleavage site^26^. Cleavage of the ADAM cleavage site by members of the ADAM/TACE (tumour necrosis factor-*α*-converting enzyme) family of metalloproteases is necessary for Notch activation^27–30^. Clearly, Delta and Notch proteins affect intercellular adhesion, and this adhesive strength in turn affects Delta-Notch signalling. Delta-Notch adhesive properties would require their respective membrane anchorage. This is contrasted with their signalling functions which would necessitate their proteolytic cleavage counteracting their adhesive roles. It is however unknown how this paradoxical interplay affects tip-stalk patterning within a system of cells. In this section, we sought to investigate how intercellular adhesion affects Notch signalling. For ease of analysis, intercellular adhesion is first considered without intracellular Notch heterogeneity, i.e. *W* = ∞. First, we use the number of adherent Delta-Notch pairs to calculate the intercellular distance between neighbouring cells. Next, using the intercellular distance calculated, the rate constant of Notch activation can be determined using Dembo’s laws^31^ which subsequently allow for elucidation of the Delta and Notch levels for each cell.

We observe that simply considering tension-dependent rate of Delta-Notch binding in lateral inhibition, we are able to generate a limited range of tip-stalk patterning. We illustrate why with the two-cell spacing patterning. The following are the system of equations for *N*_*l*,2_ and *N*_*r*,2_ of the two-cell spacing pattern at steady state when *W* = ∞. Since *W* = ∞, *N*_*l*,2_ = *N*_*r*,2_ = *N*_2_.

5 $$0=-\,{k}_{d}{N}_{2}+{k}_{f,1}({D}_{1}\mathrm{)(1}-{N}_{2})$$

6 $$0=-\,{k}_{d}{N}_{2}+{k}_{f\mathrm{,2}}({D}_{2}\mathrm{)(1}-{N}_{2})$$

Hence, by accounting for tension-dependent rate of Delta-Notch binding, a steady state two-cell spacing can be obtained provided the following constraint is fulfilled.

7 $${k}_{f\mathrm{,1}}({D}_{1})={k}_{f\mathrm{,2}}({D}_{2})$$

Examining the equations for *N*_1_ and *N*_2_ and solving for *k*_*d*_ yields the following expression for *k*_*d*_.

8 $${k}_{d}=\frac{{k}_{f1}{D}_{2}\mathrm{(1}-{N}_{1})}{{N}_{1}}=\frac{{k}_{f1}{D}_{1}\mathrm{(1}-{N}_{2})}{{N}_{2}}$$

Substituting for *D*_1_ and *D*_2_ reduces to the folowing equality

9 $$\frac{1-{N}_{1}}{{N}_{1}}[1+{(\frac{{N}_{1}}{K})}^{2}]]=\frac{1-{N}_{2}}{{N}_{2}}[1+{(\frac{{N}_{2}}{K})}^{2}]$$

**Figure 5:**
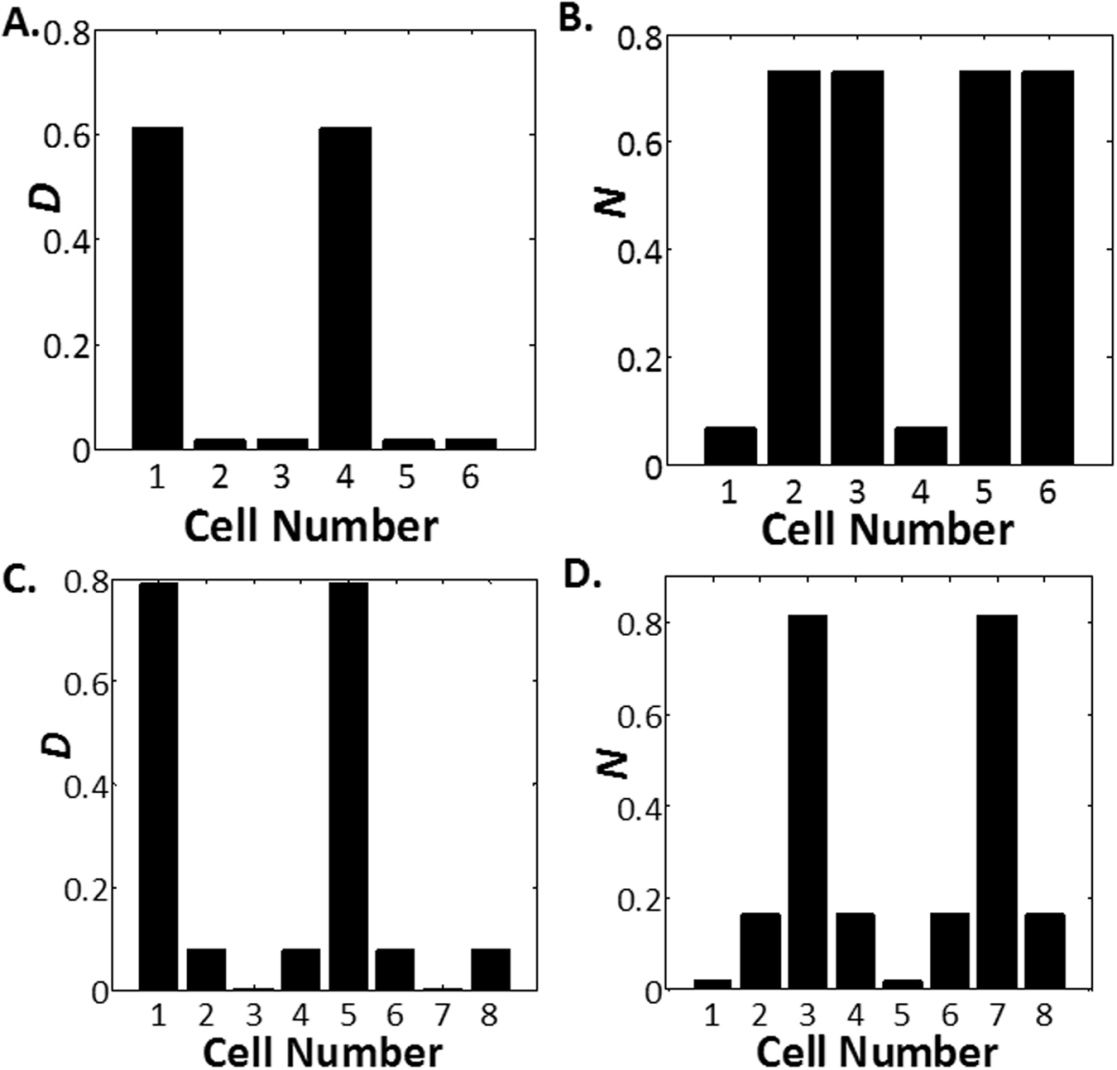
Delta and Notch Levels in Lateral Inhibition with Tension Modulation of Rate Constants. Delta levels (**A**) and Notch levels (**B**) plotted against cell number for two-cell spacing at *λ* = 30, *h* = 0.0052, *W* = ∞, *b*_0_ = 0.9, *K* = 0.1, *k*_*f*0_ = 3.719 and *k*_*d*_ = 0.0225. Delta levels (**C**) and Notch levels (**D**) plotted against cell number for three-cell spacing at *λ* = 10, *h* = 0.0761, *W* = ∞, *b*_0_ = 0.9, *K* = 0.05, *k*_*f*0_ = 38.6324 and *k*_*d*_ = 0.4021.

A trivial solution will be when *N*_1_ = *N*_2_. Such will be the case of zero-cell spacing where all cells are identical to each other. However, nontrivial solutions exist when the function *M*(*x*) = (1 − *x*)/(*x*)[1 + (*x*/*K*)^2^] is a many-to-one function. Solving for the stationary points in *M* suggests nontrivial solutions are present only when multiple positive roots exists for the function *M*′ where *M*′(*x*) = −1/*x*^2^ + 1/*K*^2^ − 2*x*/*K*^2^ = 0. This limits the parameter space in which the two-cell spacing is observed such that they can only exist if *K* < 0.1924. Furthermore, due to the necessity to fulfil the above equality, two-cell spacing is rarely observed in parameter space and only occur under a set of very narrow parameters. Similar arguments can be made for three-cell spacing. Examples of two-cell spacing and three-cell spacing are depicted in Fig. 5A,B,C,D respectively. Three-cell spacing is the maximum spacing that can be observed under tension modulation since the steady state solutions for the four-cell spacing is unstable and the function *M*′(*x*) limits the solution to a maximum of four-cell spacing.

In summary, lateral inhibition with tension-dependent rate of Delta-Notch binding allows us to reproduce some patterns but these spacings are not widely observed and only occur under a peculiar set of parameters. Nonetheless, we still manage to recover the three-cell spacing pattern which is previously not possible when we consider lateral inhibition with intracellular Notch heterogeneity.

### Lateral Inhibition with Intracellular Heteogeneity and Tension-Dependent Rate of Delta-Notch Binding Yields A Large Number of Tip-Stalk Patterns with More Than One Stalk Cell in between Tip Cells

**Figure 6:**
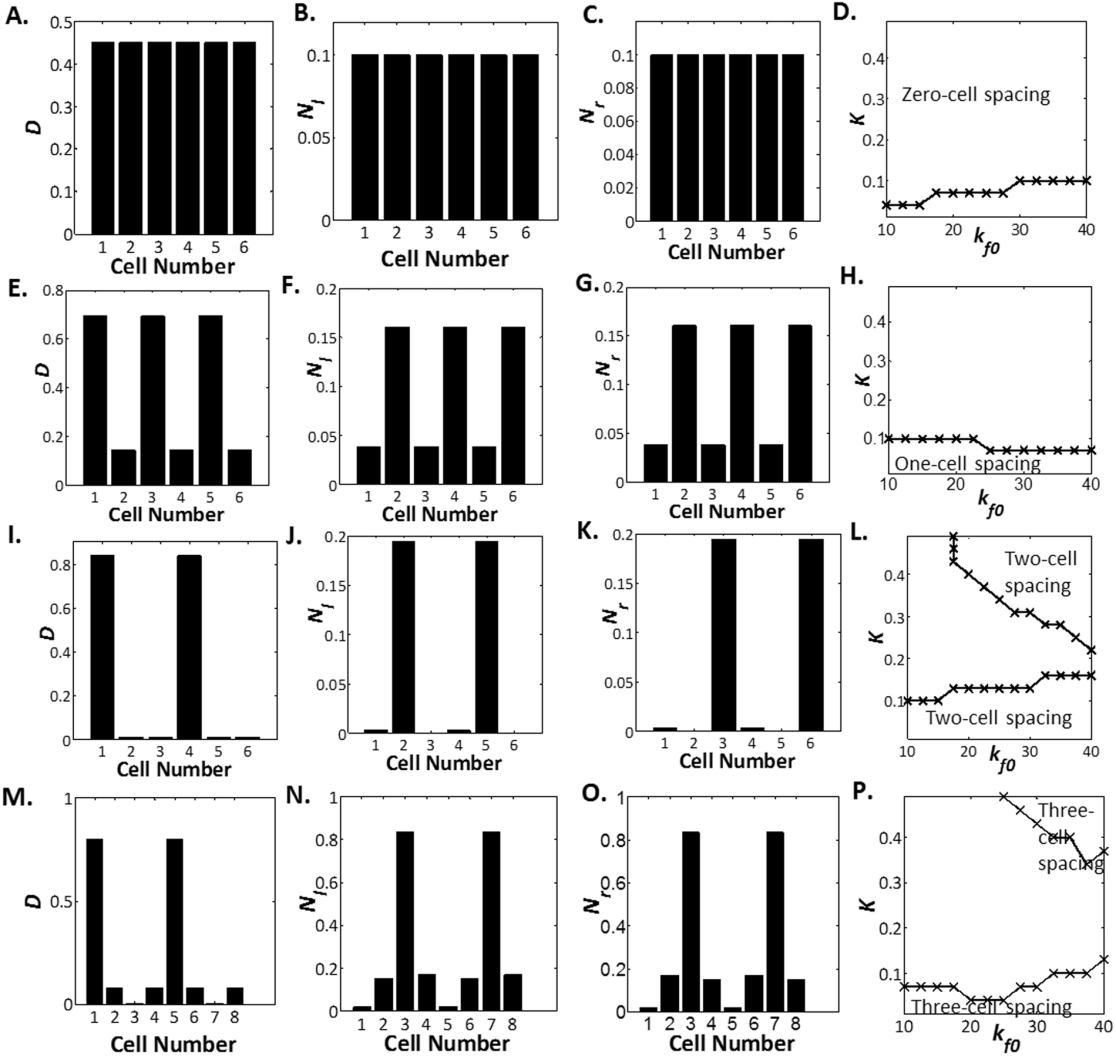
Delta and Notch Levels in Lateral Inhibition with Intracellular Notch Heterogeneity and Tension Modulation of Rate Constants. Delta levels (**A**), Notch-left levels (**B**) and Notch-right levels (**C**) plotted against cell number for zero-cell spacing at *λ* = 10, *h* = 0.076, *W* = 0, *b*_0_ = 0.9, *K* = 0.1, *k*_*f*0_ = 40 and *k*_*d*_ = 0.4. (**D**) Parameter space of *K* vs *k*_*f*0_ where zero-cell spacing is observed at *λ* = 10, *h* = 0.076, *W* = 0, *b*_0_ = 0.9 and *k*_*d*_ = 0.4. Delta levels (**E**), Notch-left levels (**F**) and Notch-right levels (**G**) plotted against cell number for one-cell spacing at *λ* = 10, *h* = 0.076, *W* = 0, *b*_0_ = 0.9, *K* = 0.07, *k*_*f*0_ = 40 and *k*_*d*_ = 0.4. (**H**) Parameter space of *K* vs *k*_*f*0_ where one-cell spacing is observed at *λ* = 10, *h* = 0.076, *W* = 0, *b*_0_ = 0.9 and *k*_*d*_ = 0.4. Delta levels (**I**), Notch-left levels (**J**) and Notch-right levels (**K**) plotted against cell number for two-cell spacing at *λ* = 10, *h* = 0.076, *W* = 0, *b*_0_ = 0.9, *K* = 0.01, *k*_*f*0_ = 40 and *k*_*d*_ = 0.4. (**L**) Parameter space of *K* vs *k*_*f*0_ where two-cell spacing is observed at *λ* = 10, *h* = 0.076, *W* = 0, *b*_0_ = 0.9 and *k*_*d*_ = 0.4. Delta levels (**M**), Notch-left levels (**N**) and Notch-right levels (**O**) plotted against cell number for three-cell spacing at *λ* = 10, *h* = 0.076, *W* = 0, *b*_0_ = 0.9, *K* = 0.01, *k*_*f*0_ = 35 and *k*_*d*_ = 0.4. (**P**) Parameter space of *K* vs *k*_*f*0_ where three-cell spacing is observed at *λ* = 10, *h* = 0.076, *W* = 0, *b*_0_ = 0.9 and *k*_*d*_ = 0.4.

Here, we present the results for the modified lateral inhibition model with intracellular Notch heterogeneity and tension-dependent rate of Delta-Notch binding in Fig. 6. We observe that the various patterns: zero-cell spacing, one-cell spacing, two-cell spacing and three-cell spacing can be attained under a large range of parameter values.

At the same time, we observe parameters where the spacings can co-exist with each other indicating the existence of a multistable steady state system. Identification of parameters where the various cell spacings can be observed may be useful in guiding experimentalists in the future as they seek to design their desired blood vasculature density. Different blood vasculature density are required for distinct biological functions. For example, during the wound healing process, a dense blood vasculature, i.e. small cell spacing, is preferred to ensure sufficient perfusion of the wounded tissue with essential materials for regrowth of the tissues. This is contrasted with tumour angiogenesis where blood vasculature can be designed to be sparser, i.e. larger cell spacing, so that the tumour becomes starved of the oxygen and nutrients it requires thus inhibiting the tumour’s growth. Knowledge of the parameter space where different spacing patterns can be easily found may allow experimentalists or tissue engineers to modify their cellular parameters such that their system of cells lie predominantly within their designed spacings.

**Figure 7:**
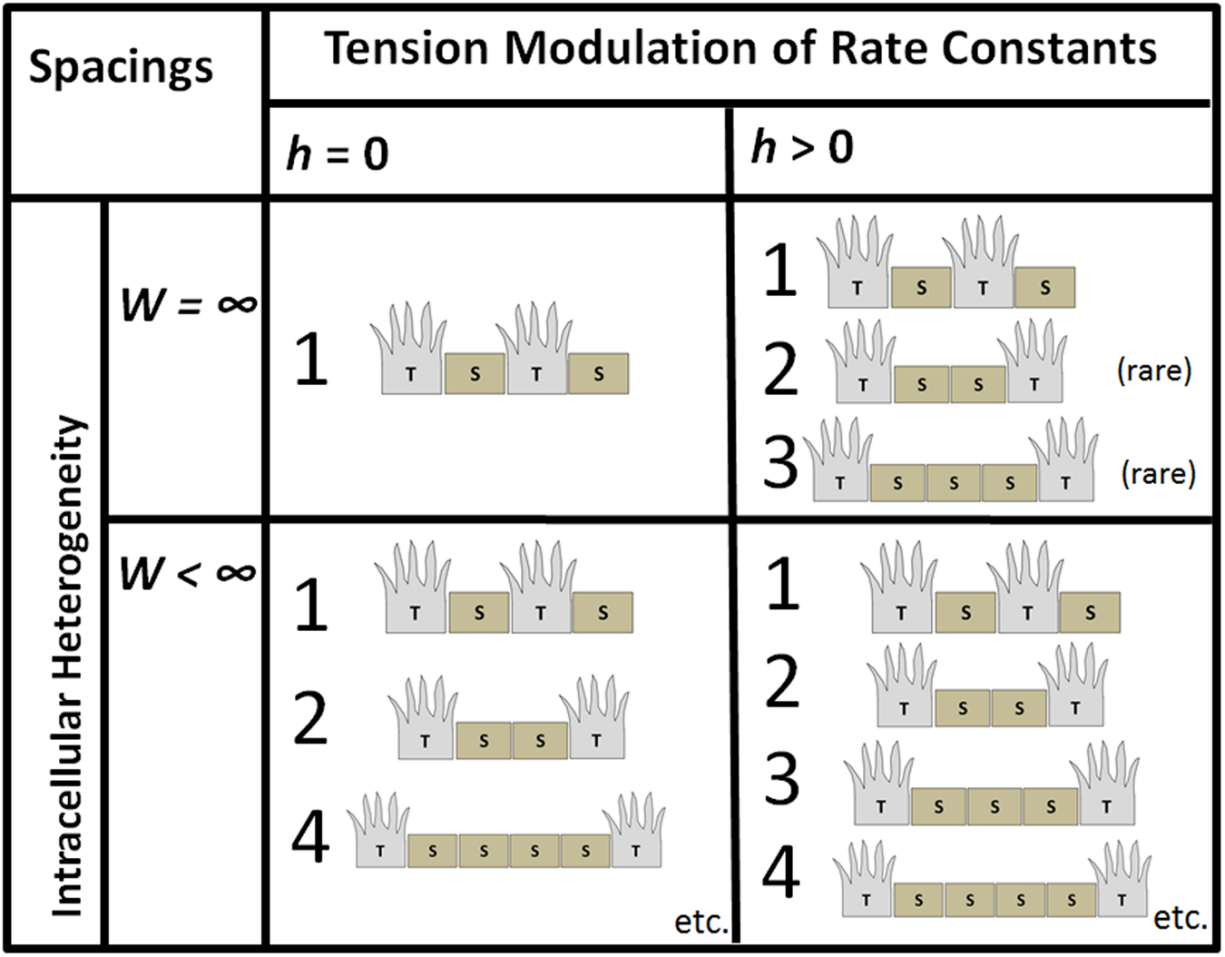
Summary of Tip-stalk Patterns Observed Under Different Conditions of *W* and *h*. Summary of tip-stalk patterns observed under different conditions of *W* and *h*. When *W* = ∞ and *h* = 0, the classical lateral inhibition model is recovered which yields exclusively the zero-cell spacing and the one-cell spacing. When *W* < ∞ and *h* = 0, this corresponds to modified lateral inhibition model with intracellular Notch heterogeneity. More tip-stalk patterns are observed such as the two-cell spacing but due to symmetry restraints, three-cell spacing is never observed. On the other hand, when *W* = ∞ and *h* > 0 which corresponds to modified lateral inhibition model with tension dependent rate of Delta-Notch binding, two-cell spacing and three-cell spacing are observed. Unfortunately, these spacings occur under very narrow parameter ranges rendering its rarity. Lastly, when *W* < ∞ and *h* > 0, which is the case of enhanced lateral inhibition model with intracellular Notch heterogeneity and tension-dependent rate of Delta-Notch binding, we recover the one-cell spacing, two-cell spacing, three-cell spacing etc. at wide parameter ranges.

Lastly, we summarise the patterns possible under different modifications to the classical lateral inhibition model in Fig. 7. It can be observed from the figure that both intracellular Notch heterogeneity and tension-dependent rate of Delta-Notch binding are necessary to recreate the various forms of tip-stalk patterns observed during angiogenesis such as two-cell spacing and three-cell spacing.

### Existence of Intermediate Cell States *In Vitro*

An important prediction in our enhanced Delta-Notch lateral inhbition model is the existence of intermediate cell states, i.e., cells that exhibit moderate Delta or Notch levels thus manifesting both tip cell and stalk cell traits. These intermediate cell states are necessary as additional building blocks besides the cannonical high Delta low-Notch tip cell and the low Delta-high Notch stalk cell to create larger spacings patterns. It has been reported that in human umbilical vein endothelial cells (HUVECs), the levels of Delta are 7 times more in CD34 expressing cells (CD34^+^) compared with CD34 negative (CD34^−^) cells. CD34^+^ cells also exhibit similar morphology and properties which are characteristic of tip cells. Therefore, CD34 have been hypothesized as a marker for tip cells^32^.

**Figure 8:**
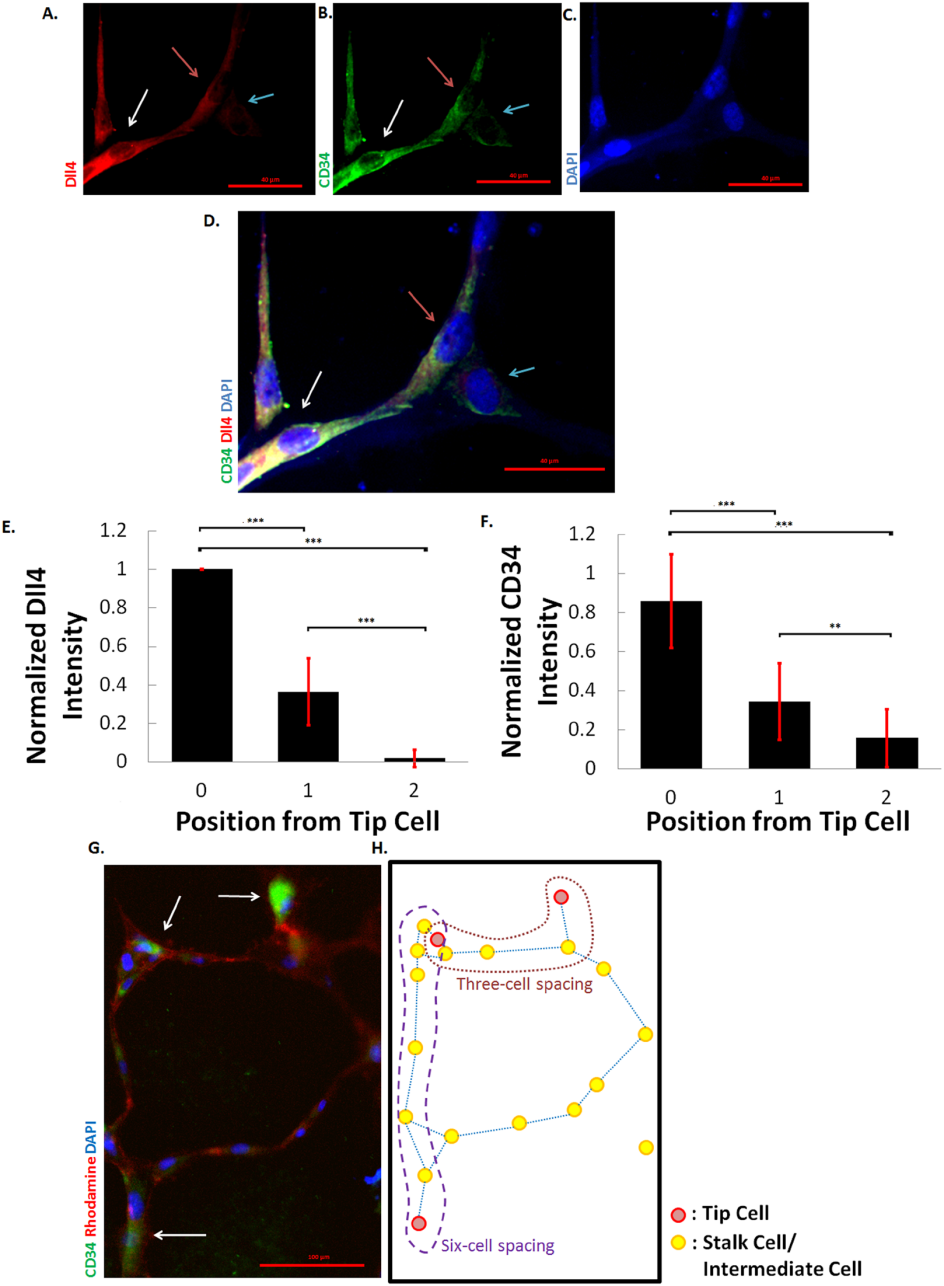
Varying Fluorescent Levels are Exhibited by Cells Depending on Their Position from the Tip Cells. HUVECs are immuno-stained using Delta or Dll4 antibody (red) (**A**), CD34 antibody (green) (**B**) and DAPI (blue) (**C**). (**D**) Overlay images of (**A**–**C**). Scale bar represents 40 *μ*m. The white arrow is pointing to a cell with a substantially brighter fluorescence as compared to its neighbours. Since Delta and CD34 are tip cell markers, the white arrow is pointing to a tip cell. The red and blue arrows are pointing to cells one-cell and two-cells away from the tip cell respectively. Scale bar represents 40 *μ*m. Normalized fluorescent intensities of the cells are plotted as a function of their position from the tip cell using Delta antibody (**E**) and CD34 antibody (**F**). For (**E**), n = 10, 12, and 12 for cells of position 0, 1 and 2 from tip cell respectively. For (**F**), n = 11, 25, and 16 for cells of position 0, 1 and 2 from tip cell respectively. Error bars denote standard deviation. *p* < 0.001 represented by *** and *p* < 0.01 represented by **. (**G**) HUVECs are immuno-stained with CD34 antibody (green), Phalloidin-rhodamine (red) and DAPI (blue). White arrows are pointing to cells that have an intense fluorescence stain for CD34. Scale bar represents 100 *μ*m. (**H**) Skeletonized image for (**G**) where red circles indicate tip cells and yellow circles represent intermediate cells and stalk cells. Physical connections between cells are represented by blue lines. The red and purple dashed lines indicate the presence of three-cell and six-cell spacing respectively.

In our current work, we used CD34 and Delta as markers for tip cells in immuno-staining experiment. We observed that cells with high(low) Delta levels also show high(low) levels of CD34 (Fig. 8A,B). Further analysis found that when each pixel in Delta staining is compared against the corresponding CD34 pixel intensity, a high correlation of 0.847 is obtained (Supplementary Fig. 2). The high correlation between CD34 and Delta supports the hypothesis that both CD34 and Delta can be used as markers for tip cells. We also observed that fluorescence intensities vary greatly amongst the cells. Strikingly, there are cells that are more brightly stained than others using Delta and CD34 antibody. Since Delta and CD34 are tip cell markers, cells that stain most strongly for these antibodies are very likely the tip cells. Furthermore, if we quantify the fluorescence intensities of the cells based on their position from the tip cell, we found that depending on their positions, cells exhibit significantly different fluorescence. This is even so if we compare fluorescence intensities of cells one-cell position away from the tip cell and cells that are two-cell position away from the tip cell as shown in Fig. 8E,F. Fluorescence intensities of cells one cell away from the tip cell exhibit significantly higher fluorescence intensities than cells two cells away from the tip cell. This observation thus suggests that we can distinguish at least three different cell types that occur during sprouting angiogenesis. The first cell type is the cell that is stained most intensely for Delta and CD34 which we designate as the tip cell. The second cell type, the stalk cell which stains the weakest for Delta and CD34. Lastly, an intermediate cell type that exhibits moderate staining. Since the validity of the model hinges on the presence of the intermediate cell, the identification of the hybrid cell thus lends evidence and weight to the legitimacy of the model.

In addition, we observe large cell spacings such as six-cell spacing in many of the stained images. An example is shown in Fig. 8G. Such large cell spacings implicate that tension modulation of rate constants is insufficient to supplement the classical lateral inhibition model to recapitulate the different spacings in nature. Observation of the three-cell spacing scenario also renders lateral inhibition with intracellular Notch heterogeneity inadequate in reproducing the various tip-stalk spacings. Based on these experimental observations, we conclude that nature operates in the regime where *W* < ∞ and *h* > 0, and that both intracellular Notch heterogeneity and tension modulation of rate constants are necessary to reproduce the myriad of tip-stalk spacings observed.

## Discussion

Lateral inhibition is a mechanism invoked in many organisms for cell fate selection. In angiogenesis, a similar cell fate selection is undertaken by endothelial cells. Lateral inhibition in angiogenesis results in endothelial cells taking on either the tip cell fate or the stalk cell fate. Classical lateral inhibition models produce the salt-and-pepper configuration where tip cells are separated exactly by one stalk cell. *In vivo* and *in vitro* experiments have however revealed a repertoire of spacing patterns not limited to the salt-and-pepper configuration such as two and three stalk cells between the tip cells. Many mechanisms have been suggested to model such patterns. However, these mechanisms may not be applicable during sprouting angiogenesis since the sprouts formed are typically one-dimensional in nature which defies Collier’s^11^ system, filopodia rarely interact with lagging stalk cells in contrast with Cohen’s^14^ and the Notch gradient term in Chen’s^15^ has not been observed. Hence, in this paper, we seek to uncover a ubiquitous lateral inhibition mechanism for recapitulating patterns observed during sprouting angiogenesis.

Classical lateral inhibition models are limited to the salt-and-pepper configuration since only two cellular states are possible: a high Delta-low Notch state and a low Delta-high Notch state. In recent years, more mechanisms that regulate Delta- Notch signalling have been uncovered. These include cis-inhibition which have been shown to increase the stability of states but not the number of states^33^. Jagged has also been identified as a crucial ligand in regulating Notch activity but Jagged expression appears to be limited; Notch and Delta are the only ligand-receptor pair expressed in capillaries^34^. As such, we look to ubiquitous and pervasive mechanisms in nature that are capable of expanding the number of states. Intracellular protein heterogeneity has been previously implicated in *Drosophila* bristle formation via the planar cell polarity mechanism^35^. To check that activated Notch can indeed be present in disparate levels within the cell, we perform immuno-staining of HUVECs with Notch antibody and image via confocal microscopy. The representative z-slice of different HUVECs is shown in Supplementary Fig. 3. As observed in Supplementary Fig. 3, different regions of the cell do exhibit different levels of fluorescence when immuno-stained with Notch antibody. Notably, if we are to divide the cell into a section with higher-Notch intensity and another section with lower-Notch intensity, the section with higher-Notch intensity is about 2 times more brightly stained than the section of the cell with lower-Notch intensity. To ensure that only activated Notch is considered, the cell membrane is excluded from the fluorescence measurements in all of the above analysis. We also ignore the top 3 z-slices and bottom 3 z-slices which correspond to the top and bottom 0.6 *μ*m which we take to be the membrane. Thus, Notch can indeed be present in heterogeneous levels within the cell. It has also been shown that tension can modulate the Notch signalling pathway and Notch may actually act as a mechanosensitive sensor^18,36^. As such, we investigate if addition of intracellular Notch heterogeneity and tension-dependent rate of Delta-Notch binding into the classical lateral inhibition model may allow us to recover the various forms of patterning observed during sprouting angiogenesis. The enhanced model with intracellular Notch heterogeneity and tension-dependent rate of Delta-Notch binding is capable of recapitulating the many forms of patterning observed such as the zero-cell spacing, one-cell spacing, two-cell spacing as well as the three-cell spacing case under a wide range of parameter values.

In recent years, hybrid cells types of various kinds have been postulated and identified. These include the hybrid epithelial/mesenchymal phenotypes^37,38^ in which co-expression of epithelial and mesenchymal signatures is strongly correlated with poor survival amongst patients suffering from breast cancer^39^. Such hybrid cell states are also observed in small cell lung cancer. In addition to the known neuroendocrine/epithelial state (NE) and the no-neuroendocrine/mesenchymal-like (ML) state, a third cell state expressing both markers of NE and ML differentiation was discovered in small cell lung cancer^40^. Clearly, hybrid cell types have important clinical consequences in therapeutic settings. In this paper, we identify the existence of a hybrid cell type that exhibit intermediary tip cell and stalk cell characteristics. The identification is based on an immuno-fluorescence stain for Delta and CD34, a known tip cell marker^32^. In the experiment, three distinct cell populations can be distinguished based on their fluorescence levels and position from the tip cells. The three cell types are the canonical tip and stalk cell, as well as the hybrid cell. The identification of this hybrid cell type is crucial to the validation of our model and is necessary as an additional building block complementing the tip cell and the stalk cell in the construction of large cell spacings. The discovery of the intermedate tip/stalk phenotype thus provides credible evidential support to the plausability of the model.

## Conclusion

In conclusion, current lateral inhibition models involving only Delta and Notch are inadequate in explaining tip-stalk pattering in sprouting angiogenesis. In this paper, we seek to uncover a general mechanism that is able to recapitulate cellular patterning observed by invoking mechanisms commonly neglected: intracellular Notch heterogeneity and tension-dependent rate of Delta-Notch binding. Such mechanisms do not require specific expression of particular genes and thus are universal across all cell types expressing Delta and Notch. We demonstrate that these two mechanisms are sufficient and necessary in recreating the rich behaviour of tip-stalk patterning observed. Furthermore, we also demarcate the parameter space for each tip-stalk pattern which may serve to guide experimentalists in the future when they seek to design their desired blood vasculature. Last but not least, we identify the existence of an intermediate cell type, a key prediction of our enhanced model thus substantiating the validity of the model as well as its prediction.

## Methods

### Experimental Procedures

#### Cell culture and Immuno-staining

Human umbilical vein endothelial cells, HUVECs were obtained from ATCC and maintained in Endothelial Growth Media, EGM-2 Bulletkit (CC-3162 and CC-4176, Lonza). Similar number of cells were grown on coverslips coated with matrigel in a six-well dish for four hours. Upon formation of capillary tubes, cells were fixed with 4% paraformaldehyde for 20 min at room temperature. The fixed cells were then permeabilized with 0.1% TritonX-100/PBS for 10 min, followed by blocking with 4% BSA/PBS 1 h at room temperature. Primary antibodies were 1:100 diluted in 1% TritonX-100/PBS and incubated with the cells at 4 °C overnight. Secondary antibodies were diluted 1:1000 in 1% TritonX-100/PBS with 1 h incubation at room temperature. Images were taken at 20× and 40× magnification with an Axio observer Z1 and PerkinElmer spinning disk confocal microscope. All immuno-staining were conducted with at least three independent experiments. The antibodies used were anti-CD34 (GeneTex and Abcam), Notch 1 (Santa Cruz), Dll4 (Abcam).

#### Image Quantification

Each cell is segmented and ImageJ is used to quantify the fluorescence intensity. Cell fluorescence intensities are normalized such that one corresponds to the brightest cell and zero corresponds to the dimmest cell in each image. The normalized cell intensities are then combined across all images and compared. The two sample t-test with unequal variance is used for normalized intensities comparison.

### Detailed Explanation of the Enhanced Delta-Notch Lateral Inhibition Model

In this section, we first describe the classical lateral inhibition model before introducing the modifications. This section will be organized as follows:

1. Classical Lateral Inhibition Model.
2. Lateral Inhibition Model with Intracellular Notch Heterogeneity.
3. Lateral Inhibition Model with Intracellular Notch Heterogeneity and Tension-Dependent Rate of Delta-Notch Binding.

Assumptions and simplifications of each modification are listed in their respective subsection. We end off the section with details on how the models are resolved computationally. In all of the equations listed below, we consider a linear one-dimensional array of cells with periodic boundary conditions. Periodic boundary conditions are used so that a long array of cells can be modelled with a tractable number of equations.

#### Classical Lateral Inhibition Model

Classical Delta-Notch lateral inhibition models take the following form: (1) Delta ligand expression is inhibited by high levels of intracellular activated Notch, and (2) Notch receptor is activated after binding of Delta expressed on neighbouring cells. At the same time, both Delta and Notch proteins undergo first order decay^11,19,20^. This feedback loop amongst neighbouring cells is embodied in the following system of equations for a linear periodic one-dimensional array of cells up to cell *N*, (1...*j*...*N*):

10 $$\frac{d{\tilde{D}}_{j}}{dt}=-\,{k}_{D}{\tilde{D}}_{j}+\frac{{B}_{0}}{1+{(\frac{{\tilde{N}}_{j}}{k})}^{2}}$$

11 $$\frac{d{\tilde{N}}_{j}}{dt}=-\,{k}_{N}{\tilde{N}}_{j}+{k}_{F}\frac{({\tilde{D}}_{j-1}+{\tilde{D}}_{j+1})}{2}({\tilde{N}}_{0}-{\tilde{N}}_{j})$$

The first equation, Eq. (10) describes the rate of change of Delta, $\tilde{D}$, in cell *j* at any time *t*. The rate of change of Delta is a combination of effects arising from Delta’s decay, contributed by the first term on the right hand side of the equation, and inhibition from activated Notch contributed by the second term. *k*_*D*_ represents the decay coefficient for Delta, *B*_0_ denotes the maximum expression rate of Delta and *k* refers to Delta’s inhibitory coefficient which is the concentration of activated Notch necessary to result in half maximal Delta expression. Since the inhibitory effect of activated Notch on Delta’s expression has been shown to follow the Hill dynamics, the Hill equation is used to model the interaction between activated Notch and Delta with a Hill coefficient of 2^41^.

Similarly, the second equation, Eq. (11) describes the rate of change of activated Notch, $\tilde{N}$ in cell *j* which is a summation of effects brought about by decay (first term on the right hand side) as well as activation by Delta from neighbouring cells, $\tilde{D}$_*j* + 1_ and $\tilde{D}$_*j*−1_. *k*_*N*_ denotes the decay coefficient of activated Notch while *k*_*F*_ is the rate constant of the binding reaction between Delta and inactive Notch where $\tilde{N}$_0_ represents the total amount of activated and inactivated forms of Notch.

In nondimensional form, the system of equation reduces to the following:

12 $$\frac{d{D}_{j}}{d\tau }=-\,{D}_{j}+\frac{{b}_{0}}{1+{(\frac{{N}_{j}}{K})}^{2}}$$

13 $$\frac{d{N}_{j}}{d\tau }=-\,{k}_{d}{N}_{j}+{k}_{f}({D}_{j-1}+{D}_{j+1}\mathrm{)(1}-{N}_{j})$$

where $$t=\frac{\tau }{{k}_{D}}$$, $$\tilde{D}$$_*j*_ = *D*_0_*D*_*j*_, $${D}_{0}=\frac{{k}_{d}}{{B}_{0}}$$, $$\tilde{N}$$_*j*_ = *N*_0_*N*_*j*_, $${b}_{0}=\frac{{B}_{0}}{{k}_{D}{D}_{0}}$$, $$K=\frac{k}{{N}_{0}}$$, $${k}_{f}=\frac{{K}_{f}{D}_{0}}{2{k}_{D}}$$ and $${k}_{d}=\frac{{k}_{N}}{{k}_{D}}$$.

In general, a lower *K* and higher *k*_*f*_ implies greater nonlinearity within the system of equations. A low *K* signifies a low concentration of activated Notch necessary for maximal inhibition of Delta while a high *k*_*f*_ indicates a low level of Delta necessary for activation of the neighbouring Notch receptor.

#### Lateral Inhibition Model with Intracellular Notch Heterogeneity

Modelling the above system of equations namely Eqs (12) and (13) on a one-dimensional grid of cells will obtain the salt-and-pepper steady state configuration where tip cells are regularly spaced by one stalk cell. Inherent in this system of equations is the assumption that Notch levels are homogeneous throughout the cell. Notch is activated by Delta expressed on its neighbours and should the Delta levels of the neighbours differ, the amount of Notch that is activated within different parts of the cell may also be different. As such, Notch protein may not necessarily be homogeneous throughout the cell. Intracellular heterogeneity has previously being implicated in *Drosophila* bristle formation via the planar cell polarity pathway^35,42^. In^42^, a negative feedback loop couples adjacent sides of neighbouring cells. If the negative feedback loop is sufficiently strong, individual cells willl polarize and exhibit disparate concentration of proteins along different regions of the cell. Ultimately, this leads to an entire cell sheet possessing polarity. It is currently unknown how intracellular heterogeneity will affect patterning during sprouting angiogenesis. To include intracellular heterogeneity of activated Notch levels into lateral inhibition, we modify the above system of equations to account for differential levels of activated Notch within the cell. For simplicity, we adopted a similar approach in^42^ and modelled each cell as having two sides where *N*_*l*,*j*_ and *N*_*r*,*j*_ represents fraction of activated Notch on the left and right side of cell *j* respectively.

Notch is activated by Delta expressed on neighbouring cells. Thus depending on the concentration of Delta in its neighbours, a cell may possess different levels of activated Notch where the side of the cell with a higher Delta neighbour will have higher levels of activated Notch, or NICD. Delta is however inhibited by the overall levels of activated Notch within the cell and thus is unlikely to exhibit deviating levels intracellularly. Hence, in the following modified lateral inihibition model, we only consider intracellular Notch heterogeneity.

Figure 2B depicts the schematic for lateral inhibition after considering for intracellular Notch heterogeneity with the system of equations listed below.

14 $$\frac{d{D}_{j}}{d\tau }=-\,{D}_{j}+\frac{{b}_{0}}{1+{(\frac{\frac{{N}_{l,j}+{N}_{r,j}}{2}}{K})}^{2}}$$

15 $$\frac{d{N}_{l,j}}{d\tau }=-\,{k}_{d}{N}_{l,j}+{k}_{f}({D}_{j-1}\mathrm{)(1}-{N}_{l,j})+W({N}_{r,j}-{N}_{l,j})$$

16 $$\frac{d{N}_{r,j}}{d\tau }=-\,{k}_{d}{N}_{r,j}+{k}_{f}({D}_{j+1}\mathrm{)(1}-{N}_{r,j})+W({N}_{l,j}-{N}_{r,j})$$

where *W* = *F*_*Notch*_/(*L*^2^*k*_*D*_*N*_0_) in which *F*_*Notch*_ is the diffusion coefficient of Notch and *L* is the length of the cell.

Like the classical lateral inhibition model, Eq. (14) describes how decay of Delta and Notch inhibition affects the rate of change of Delta. A slight difference exists between Eqs (14) and (12). In Eq. (14), Delta is inhibited by the average levels of Notch within the cell. This is however unnecessary in Eq. (12) due to the assumption of Notch homogenity. At the same time, the original equation for Notch, Eq. (13) is split into two separate equations, one for each side of the cell where Eqs (15) and (16) describes the rate of change of activated Notch on the left and right side of the cell respectively. As in^42^, a separate term that accounts for the exchange of Notch between the left and right side of the cells is introduced into the right hand side of Eqs (15) and (16). This exchange is characterized by a diffusion term, *W*. When *W* goes to infinity representing extremely fast diffusion, activated Notch levels on the left and right side of the cell equilibrates and the classical lateral inhibition model is recovered.

In order to determine the validity of the assumption of Notch heterogeneity, time taken for diffusion of Notch was compared against time taken for reaction. Mean diffusion coefficient of Notch has been determined to be in the order of 0.076 *μ*m^2^/s using quantum dots^43^. The diffusion rate of Notch translates into a mean diffusion time of 5000 s in a cell of 10 *μ*m radii^44^. Comparatively, this diffusion time is slow in comparison to typical ligand binding reactions undertaken during Notch activation upon Delta binding where a prototypical ligand induced conformation change takes 1 *ms*^45^.

#### Lateral Inhibition Model with Intracellular Notch Heterogeneity and Tension-Dependent Rate of Delta-Notch Binding

Receptor-ligand binding reactions have been known to be dependent on the distance between receptor and ligand^46,47^. Intuitively, if the receptor and ligand are too far apart, successful binding cannot take place. On the other hand, if they are too close, steric hindrance may interfere with binding. This dependence on receptor-ligand distance is incorporated in Bell’s model where adhesion between cells and substratum is modelled by allowing the bond association and dissociation rates between the cell receptor and substrate ligand to vary as an exponential function of their bond length^48^. Notch activation has also been shown to be dependent on receptor-ligand distance. By utilizing atomic force microscopy on live cells, Ahimou *et al*. established that the intercellular adhesive force affects the rate of Notch signalling where Delta pulling promotes Notch activation^18^. Furthermore, the Notch pathway has recently being implicated in tension-regulation of cells where components of the Notch signalling pathway are postulated to respond to low mechanical tension resulting in the inhibition of h2-calponin expression^36^. Therefore, there is prevailing evidence that suggests Delta-Notch signalling is influenced by intercellular tension. Unfortunately, to our best knowledge, it is not known how mechanical forces impacts Delta-Notch signalling within a system of cells and consequently how this tension contributes to tip-stalk patterning.

To account for how bond stress affects bond strain and ultimately the rate constants between adhesive molecules, Dembo and coworkers introduced a set of constitutive laws to calculate the chemical kinetics of the adhesion molecules^31^. As such, we modified the rate constant, *k*_*f*_ within Eqs (15) and (16) by allowing it to vary according to Dembo’s model such that

17 $${k}_{f,j}={k}_{f0}\exp (-\frac{{\sigma }_{ts}{({x}_{m,j}-\lambda )}^{2}}{2{k}_{b}T})$$

In Eq. (17), *k*_*f*,*j*_ varies as an exponential function of the distance between neighbouring cells, *x*_*m*,*j*_ and the optimum distances between neighbouring cells, *λ*. Here, *k*_*f*,*j*_ represents the rate constant of the Notch activation reaction between cells *j* and *j* + 1, *x*_*m*,*j*_ denotes the intercellular distance between cell *j* and *j* + 1, *k*_*f*0_ represents the initial reaction rates, *k*_*b*_ is the Boltzmann constant, *σ*_*ts*_ is the spring constant of the transition state while *T* is the temperature. Eq. (17) dictates that at optimum bond length between receptor and ligand, the rate constant will be the highest while deviations away from the optimum bond length will cause the rate constant to decrease.

Distances between neighbouring cells are calculated by considering the number of adherent Delta and Notch pairs at the cell-cell interface and allowing the intercellular distance to be a monotically decreasing function of adherent molecules.

18 $${x}_{m,j}=F({N}_{r,j},{D}_{j+1},{N}_{j+1},{D}_{j})$$

We use the following function for *F*, where

19 $$F({N}_{r,j},{D}_{j+1},{N}_{j+1},{D}_{j})=\frac{1}{max\mathrm{([(1}-{N}_{r,j}),{D}_{j+1}])+max\mathrm{([(1}-{N}_{l,j+1}),{D}_{j}])}$$

Grouping constants together result in the final form of equation that describes *k*_*f*,*j*_

20 $${k}_{f,j}={k}_{f0}\exp (\,-\,h{(F({N}_{r,j},{D}_{j+1},{N}_{j+1},{D}_{j})-\lambda )}^{2})$$

where *h* characterizes how the rate constants vary as a function of number of adherent Delta-Notch pairs. *h* > 0 implies tension-dependent rate of Delta-Notch binding while *h* = 0 suggests that the binding rate constants are independent of number of Delta-Notch adherent pairs. Figure 2C outlines the simplified representation for lateral inhibition with both intracellular Notch heterogeneity and tension-dependent rate of Delta-Notch binding.

To summarise, the classical lateral inhibition model comprises of Eqs (12) and (13) while the enhanced lateral inhibition model consists of Eq. (14) to Eq. (20).

These equations are then solved using Matlab to find the roots to the coupled system of equations at steady state. More details can be found in the Supplementary Information. We denote *n*-cell spacing as *n* number of stalk cells between the tip cells and tip cells are defined as cells with the highest Delta concentration while stalk cells consists of cells with lower Delta concentration.

### Data Availability

No datasets were generated or analysed during the current study.

## Electronic supplementary material

Supplementary Information
