# Supplementary material for: Enhanced Delta-Notch Lateral Inhibition Model Incorporating Intracellular Notch Heterogeneity and Tension-Dependent Rate of Delta-Notch Binding that Reproduces Sprouting Angiogenesis Patterns
Source: Sci Rep. 2018 Jun 22;8:9519. doi: 10.1038/s41598-018-27645-1 (PMC6015056; doi:10.1038/s41598-018-27645-1)
Supplement: Supplementary file 1 — Supplementary Information [file 41598_2018_27645_MOESM1_ESM.pdf]

Enhanced Delta-Notch Lateral Inhibition Model Incorporating Intracellular Notch Heterogeneity and Tension-Dependent Rate of Delta-Notch Binding that Reproduces Sprouting Angiogenesis Patterns

Yen Ling Koon, Songjing Zhang, Muhammad Bakhait Rahmat, Cheng Gee Koh, Keng-Hwee Chiam

Supplementary Information

Solving the equations

We present how we derive the solution for the two-cell spacing case analytically when W and h are kept at 0. When W and h are not zero, the matlab function *fsolve* is used to probe for existence of solutions.

We first consider a linear periodic array of cells and assume the existence of a two-cell spacing pattern. Our role then is to determine if solutions exists for the two-cell spacing case and if the solutions are stable. Since it is unlikely for Delta-Notch lateral inhibition to cause left-right assymmetry in an array of cells, we limit our solutions to cases where left-right symmetry exists. Due to symmetry, $D_3 = D_2$, $N_{r,3} = N_{l,2}$ and $N_{r,2} = N_{l,3} = N_{l,1} = N_{r,1} = N_1$. The modified system of equations reduces to the following:

$$\frac{dD_1}{d\tau} = -D_1 + \frac{b_0}{1+(\frac{N_1}{K})^2} \tag{1}$$

$$\frac{dN_1}{d\tau} = -k_d N_1 + k_f D_2 (1-N_1) \tag{2}$$

$$\frac{dD_2}{d\tau} = -D_2 + \frac{b_0}{1+(\frac{N_1+N_{l,2}}{2K})^2} \tag{3}$$

$$\frac{dN_{l,2}}{d\tau} = -k_d N_{l,2} + k_f D_1 (1-N_{l,2}) \tag{4}$$

At steady state, rate of change reduces to 0 resulting in the following equalities

$$D_1 = \frac{b_0}{1+(\frac{N_1}{K})^2} \tag{5}$$

$$N_1 = \frac{k_f D_2}{k_d + k_f D_2} \tag{6}$$

$$D_2 = \frac{b_0}{1+(\frac{N_1+N_{l,2}}{2K})^2} \tag{7}$$

$$N_{l,2} = \frac{k_f D_1}{k_d + k_f D_1} \tag{8}$$

Substituting $N_{l,2}$ and D_2, we get

$$D_1 = \frac{b_0}{1+(\frac{N_1}{K})^2} = Y(N_1) \tag{9}$$

$$D_1 = \frac{k_d(2K\sqrt{\frac{b_0 k_f(1-N_1)}{k_d N_1}} - 1 - N_1)}{k_f(1-2K\sqrt{\frac{b_0 k_f(1-N_1)}{k_d N_1}} - 1 - N_1)} = Z(N_1) \tag{10}$$

Hence, depending on the values of the parameters used, the two-cell spacing scenario exists when $Y(N_1) = Z(N_1)$.

Stability analysis is then performed after the roots has been obtained to check for stability. The Jacobian matrix of the system of equations Supplementary Eq 1, Supplementary Eq 2, Supplementary Eq 3 and Supplementary Eq 4 is computed using the roots computed at steady state. Roots are stable if and only if the sign of real part of eigenvalues of the Jacobian matrix is negative. Only stable steady states are reported in the Results section.

For larger cell spacings in which analytical solutions are not possible, random initial conditions are used and the matllab function *fsolve* is used to solve for solutions. Like before, solutions are checked for stability and only stable steady state solutions are reported.

Finite diffusion leads to stable steady state solution for two-cell spacing for Lateral Inhibition Model with Intracellular Notch Heterogeneity

In this section, we present why as long as diffusion remains finite, it is always possible to have a stable steady state solution for the two-cell spacing for Lateral Inhibition Model with Intracellular Notch Heterogeneity.

For convenience of analysis, we limit our solutions to cases where left-right symmetry exists in a linear array of cells. Due to symmetry, the modified system of equations reduces to the following at steady state,

$$N_{r,2} = N_{l,2} + \frac{k_d N_{l,2} - k_f(D_1)(1-N_{l,2})}{W} = N_{l,2} + \alpha \tag{11}$$

where α denotes the difference between $N_{l,2}$ and $N_{r,2}$. α goes to zero only when W goes to infinity.

Substituting α yields

$$D_1 = \frac{k_d N_{l,2} - W\alpha}{k_f(1-N_{l,2})} \tag{12}$$

$$D_2 = \frac{k_d N_{l,2} + W\alpha + \alpha k_d}{k_f(1-N_{l,2}-\alpha)} \tag{13}$$

Hence, D_2 will be different from D_1 so long as α does not become zero. This means that as long as W is finite, the two-cell spacing pattern exists regardless of the value of W.

Examples of four-cell spacing for Lateral Inhibition Model with Intracellular Notch Heterogeneity and Tension-Dependent Rate of Delta-Notch Binding

In this section, we present examples of four-cell spacing for Lateral Inhibition Model with Intracellular Notch Heterogeneity and Tension-Dependent Rate of Delta-Notch Binding as illustrated in Supplementary Figure 1.

Validation of CD34 as a proxy for Delta

To confirm that CD34 can indeed be used a proxy as Delta in our experiments, we perform coimmuno-staining of CD34 and Delta on HUVECs. Next, we perform a correlation of each pixel's Delta intensity against the corresponding CD34 pixel intensity and observe a high correlation of 0.847. The high correlation between CD34 and Delta reinforces the notion that CD34 intensity is associated with Delta and that CD34 and Delta are associated. In Supplementary Figure 2, the pixel intensities of the cells labelled by white, red and blue arrows in Figure 8A are represented by red, green and black dots respectively.

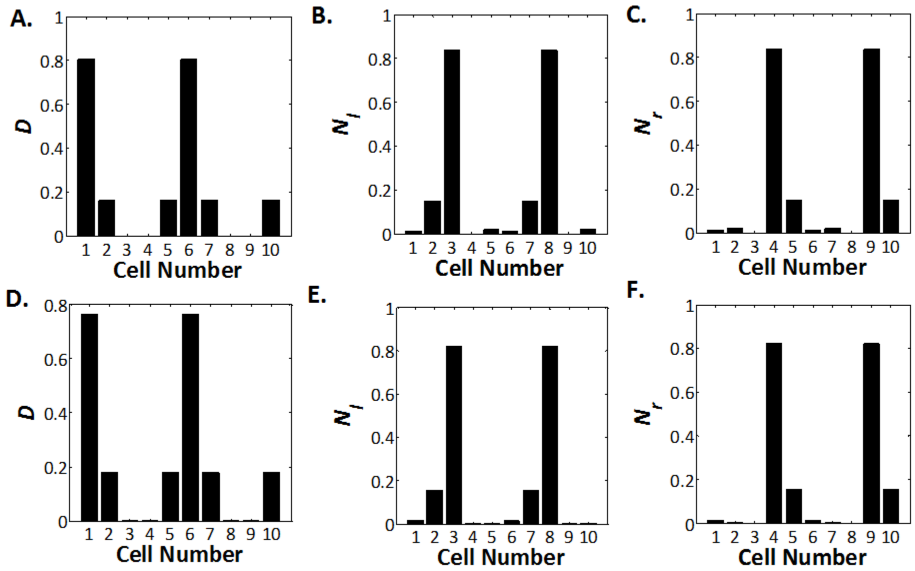

Figure 1. Four-cell Spacing in Lateral Inhibition with Intracellular Notch Heterogeneity and Tension-Dependent Rate of Delta-Notch Binding. Delta levels (A), Notch-left levels (B) and Notch-right levels (C) plotted against cell number for four-cell spacing at $\lambda = 10$, $h = 0.076$, $W = 0$, $b_0 = 0.9$, $K = 0.04$, $k_{f0} = 40$ and $k_d = 0.4$.
Delta levels (D), Notch-left levels (E) and Notch-right levels (F) plotted against cell number for four-cell spacing at $\lambda = 10$, $h = 0.076$, $W = 0$, $b_0 = 0.9$, $K = 0.04$, $k_{f0} = 45$ and $k_d = 0.4$

Validation of heterogeneity of Notch in HUVECs

In order to validate that Notch levels may not be homogenous in the cell, we perform immuno-staining of HUVECs with Notch antibody and image using confocal microscopy. The representative z-slice of different HUVECs is shown in Supplementary Figure 3A, D and G. By using a z-slice for analysis, we are able to isolate the fluorescent intensities of the cell interior from the cell surface. As inactivated Notch is present at the cell membrane, by only considering the fluorescence from the cell interior, what we then observe is the presence of activated Notch and not inactivated Notch. As observed in these images, Notch exhibits different fluorescent intensity intracellularly. Notably, if we are to divide the cell into the section of the cell with higher-Notch intensity, "Higher-Notch" (demarcated by pink line) and the section of the cell with lower-Notch intensity, "Lower-Notch" (demarcated by yellow line), the section of the cell with higher-Notch intensity is about 2 times more brightly stained than the section of the cell with lower-Notch intensity. This is demonstrated in Supplementary Figure 3C, F and I. To ensure that only activated Notch is considered, the cell membrane is excluded from the fluorescence measurements in all of the above analysis. We also ignore the top 3 z-slices and bottom 3 z-slices which correspond to the top and bottom 0.6 μm which we take to be the membrane. Thus, activated Notch can indeed be present in heterogeneous levels within the cell.

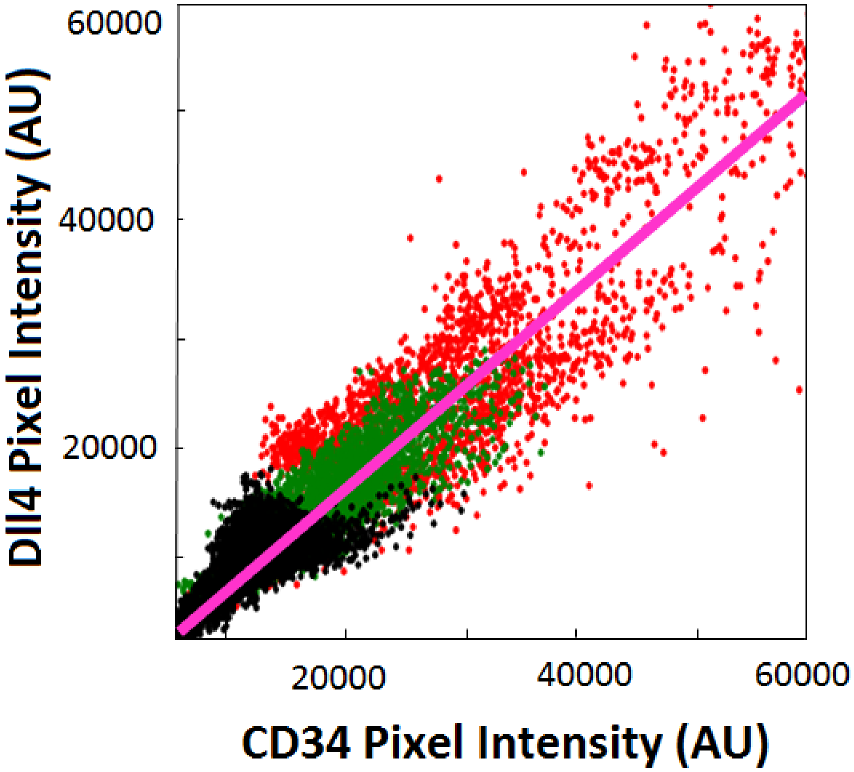

Figure 2. CD34 is a Proxy for Delta. CD34 pixel intensity is plotted againt its corresponding Dll4 pixel intensity for cells identified in Figure 8A. Pixel intensities of the cells labelled by white, red and blue arrows are represented by red, green and black dots respectively. The best fit line is plotted as a magenta line.

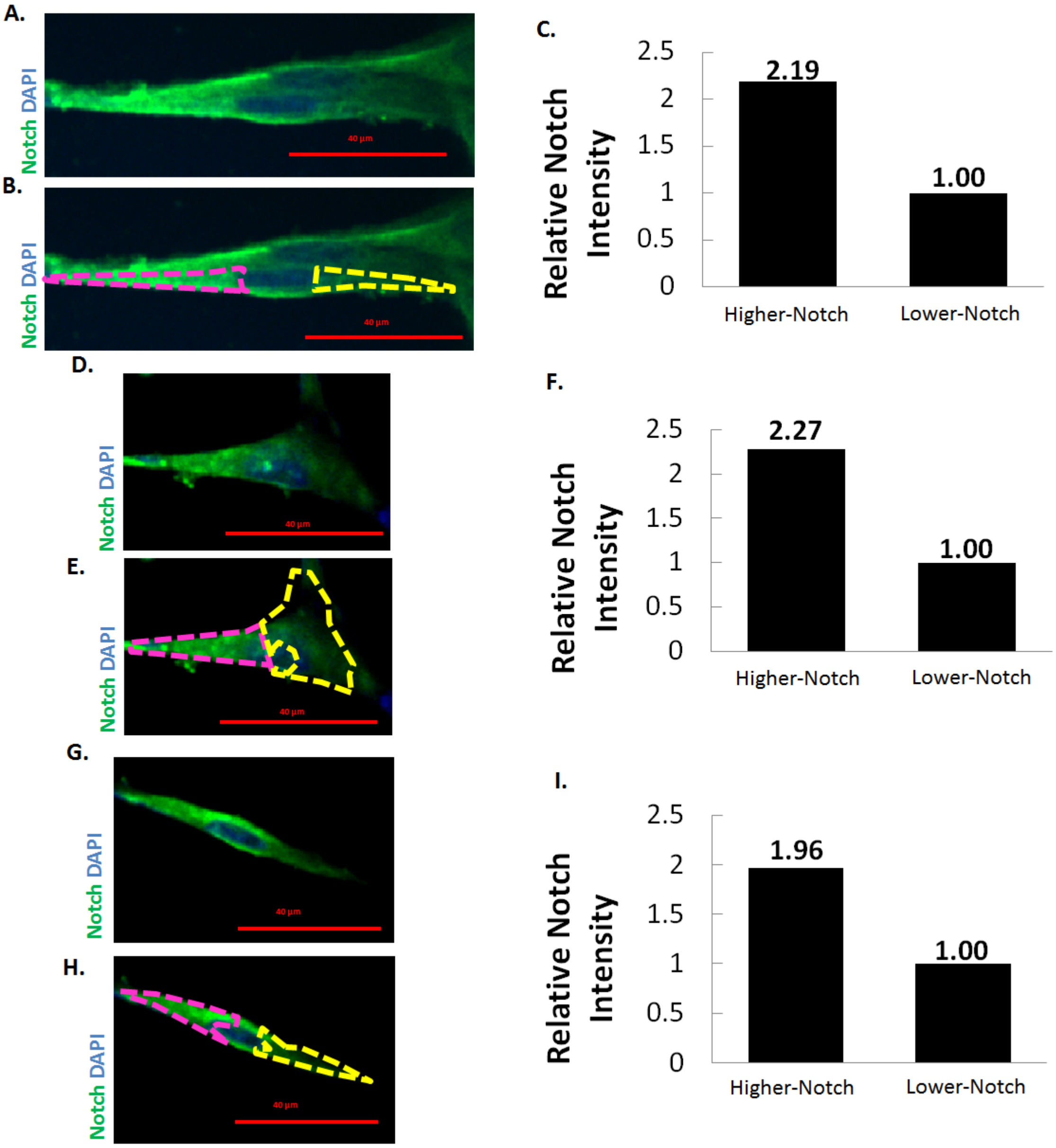

Figure 3. Observation of Notch Heterogeneity in HUVECs. (A, D, G) Representative z-slice of HUVECs immuno-stained with Notch antibody (green) and DAPI marker (blue) imaged via confocal microscopy. A, D, G are obtained at 2.4μm, 3.4μm and 3.0μm from the substrate. (B, E, H) The cell is demarcated into the section with higher-Notch fluorescence, "Higher-Notch" (pink boundary) and the section with lower-Notch fluorescence, "Lower-Notch" (yellow boundary). Scale bar represents 40μm. (C, F, I) Comparison of relative Notch fluorescence intensity levels between the section with higher-Notch fluorescence and the section with lower-Notch fluorescence for cells in (B, E, F). The cell membrane is excluded from the fluorescence measurements in the above analysis. The z-slices shown here are between 0.6μm from the bottom of the substrate and 0.6μm from the top of the cells to ensure that fluorescence from Notch residing within the cell membrane is not considered.
